# Efficient Hydroxyapatite Extraction from Salmon Bone Waste: An Improved Lab-Scaled Physico-Chemico-Biological Process

**DOI:** 10.3390/molecules29174002

**Published:** 2024-08-24

**Authors:** Francisco Muñoz, Ziyad S. Haidar, Andreu Puigdollers, Ignacio Guerra, María Cristina Padilla, Nicole Ortega, Mercedes Balcells, María José García

**Affiliations:** 1 Facultad de Odontología, Universidad Internacional de Cataluña, 08029 Barcelona, Spain; 2 Laboratorio BioMAT’X R&D&I (HAiDAR I+D+i LAB), Universidad de los Andes, Santiago 7550000, Chile; 3 Centro de Investigación e Innovación Biomédica (CiiB), Universidad de los Andes, Santiago 7550000, Chile; 4 Programa de Doctorado en BioMedicina, Facultad de Medicina, Universidad de los Andes, Santiago 7550000, Chile; 5 Programa de Doctorado en Ciencias Odontológicas, Facultad de Odontología, Universidad de los Andes, Santiago 7550000, Chile; 6 Facultad de Odontología, Universidad de los Andes, Santiago 7550000, Chile; 7 Área de Ortodoncia, Facultat Internacional de Cataluña, 08195 Barcelona, Spain; 8 Laboratorio de Investigación e Ingeniería de Biopolímeros (BiopREL), Universidad de los Andes, Santiago 7550000, Chile; 9 Escuela de Nutrición y Dietética, Facultad de Medicina, Universidad de los Andes, Santiago 7550000, Chile; 10 Institut Quimic de Sarria, Ramon Llull University, 08017 Barcelona, Spain; merche@mit.edu; 11 MIT Institute for Medical Engineering and Science, Massachusetts Institute of Technology, Cambridge, MA 02139-4307, USA

**Keywords:** hydroxyapatite, salmon bone, waste, bone repair, biomaterial, osseoregenerate, process

## Abstract

The demand for novel tissue grafting and regenerative wound care biomaterials is growing as traditional options often fall short in biocompatibility, functional integration with human tissue, associated cost(s), and sustainability. Salmon aquaculture generates significant volumes of waste, offering a sustainable opportunity for biomaterial production, particularly in osteo-conduction/-induction, and de novo clinical/surgical bone regeneration. Henceforth, this study explores re-purposing salmon waste through a standardized pre-treatment process that minimizes the biological *waste* content, followed by a treatment stage to remove proteins, lipids, and other compounds, resulting in a mineral-rich substrate. Herein, we examined various methods—alkaline hydrolysis, calcination, and NaOH hydrolysis—to better identify and determine the most efficient and effective process for producing bio-functional nano-sized hydroxyapatite. Through comprehensive chemical, physical, and biological assessments, including Raman spectroscopy and X-ray diffraction, we also optimized the extraction process. Our modified and innovative alkaline hydrolysis–calcination method yielded salmon-derived hydroxyapatite with a highly crystalline structure, an optimal Ca/P ratio, and excellent biocompatibility. The attractive nano-scale cellular/tissular properties and favorable molecular characteristics, particularly well-suited for bone repair, are comparable to or even surpass those of synthetic, human, bovine, and porcine hydroxyapatite, positioning it as a promising candidate for use in tissue engineering, wound healing, and regenerative medicine indications.

## 1. Introduction

Despite the availability of various FDA-approved bone regeneration therapies—including autografts, allografts, and synthetic bone grafts, alongside the recent and continuously accruing advancements in growth factors and bioactive molecules—there remains an ongoing demand for more innovative, efficient, effective, malleable, and sustainable solutions [1]. This need is driven by the search for alternatives that match the quality of autografts in terms of osteoconductivity, osteoinductivity, and osteogenicity. Indeed, in the realm of modern medicine and dentistry, the quest to address simple and complex bone defects arising from a myriad of causes, including disease and trauma, stands as a paramount and growingly demanding challenge [1,2]. Today, this collective endeavor has spurred extensive research into the development of novel biomaterials tailored for bone (and soft tissue) repair and regeneration, in particular, with a profound impact on our day-to-day clinical and surgical practice [2,3]. It is imperative to remember and comprehend that in the landscape of bone grafting techniques, given the diverse array of patients, each presents unique clinical and surgical needs [1,2,3,4]. Thus far, bone grafts can be categorized into four primary groups—autografts, allografts, allografts/alloplastics, and xenografts, each bearing distinct properties tailored to specific indications and applications: osteoconduction, osteoinduction, osteogenesis, and structural support. In fact, the pursuit of safe and effective bone grafting solutions is driven by a substantial demand, with millions (statistically estimated at >2 million cases in the United States of America, alone) of bone graft procedures performed globally every year (*a substantially costly burden*) [1,4,5].

Briefly, autografts have long been regarded as the ‘*gold standard*’ for de novo bone regeneration due to their superior histocompatibility and all-encompassing characteristics as an *ideal* hard tissue graft. They offer osteoinduction, osteoconduction, and cellular histocompatibility [1,2,3]. However, they are not without their limitations, as they often entail extensive morbidity associated with the donor site, necessitating longer and more expensive hospital admissions than the grafting procedure itself [2,3,4,5]. Allografts, depending on their production methods, serve as osteoconductive biomaterials or even exhibit osteoinductive properties [1,4,6]. Their increased availability compared to autografts positions them as viable alternatives; however, they often fall short in achieving full regeneration and are scrutinized for the potential transmission of viruses and other infectious agents [7]. Also, some religious beliefs preclude the use of allografting [1,2,3,6]. In contrast, synthetic alloplastic materials, while lacking osteogenic and osteoinductive potential, excel in promoting osteoconduction by serving as a scaffold and/or matrix for hard tissue regeneration and repair. Herein, the level of porosity, re-absorption capacity, and crystallinity, pivotal features of biomaterials, hinges predominantly on the manufacturing and production process(es) [1,8]. Lastly, xenografts, devoid of intrinsic osteogenic potential, face varying levels of acceptance depending on the species they originate (or, are sourced) from, with some faiths/religions, traditions, customs, and cultures opposing their use [9]. Despite these limitations, shortcomings, and/or drawbacks, scaffold osteoconduction remains effective and widely available and acceptable, circumventing the limiting complications often associated with the use of allografts and autografts [8], whilst awaiting better alternatives.

Hydroxyapatite (HA) is widely utilized in tissue regeneration and repair due to its exceptional biocompatibility, bioactivity, and structural similarity to the mineral components of human bone [1,2,4,6]. Various types of HA can be obtained from multiple sources through diverse extraction methods, each influencing the characteristics and performance of the resulting material in vitro, in vivo, and in the clinic [1,2,6,8]. Briefly, human-derived HA closely mimics autografts in functionality, offering superior biocompatibility and bioactivity; however, its application is constrained by limited availability and ethical concerns. Synthetic HA provides high purity and customizable properties; however, it may lack the innate bioactivity found in biological sources. On the other hand, whilst bovine- and porcine-derived HA offer a natural architecture and porosity that facilitate cell growth and tissue integration, they pose potential risks of disease transmission and immunogenic responses [1,2,3,4,5,6,8]. Fish-derived HA, particularly from salmon, presents a sustainable alternative with desirable nanoscale features and high bioactivity, making it a compelling candidate for tissue regeneration applications [1,2,3,4,5,6,8,9,10,11,12]. Consequently, it is noteworthy that the selection of a suitable HA type depends on specific clinical requirements, desired material properties, and ethical considerations, as each variant presents its own set of pros and cons. Indeed, in the domain of bioceramic materials, diversity reigns, contingent upon the specific species of origin from which they are sourced. To uncover the optimal characteristics while simultaneously streamlining production costs, relentless efforts have been invested in the production and creation of osteoconductive and osteoinductive biomaterials derived from various species, including but not limited to bovine, porcine, mollusks, and fish [6,7,8,9,10]. Within this perpetual quest, a notable breakthrough has recently emerged—namely, the isolation, extraction, and production of HA from the bones of salmon fish (*waste*), as aforementioned, offering a two-fold advantage: sustainability and biocompatibility. Indeed, the salmon industry predominantly focuses on the commercialization of salmon fillets, resulting in a surplus of by-products, encompassing heads, entrails, scales, and bones, which are often disproportionately discarded into the environment, causing ecological (eco-system) harm/damage [11,12,13,14,15]. This surplus of waste represents a *novel* opportunity for sustainable waste management and re-purposing. Notably, R&D&I (research, development and innovation) findings have thus far indicated that this HA bioceramic material demonstrates impressive biocompatibility, as evidenced by in vitro tests revealing an absence of cytotoxicity [12]. Moreover, Shi et al. underscored an additional advantage, wherein salmon-derived HA is enriched with vital mineral ions that foster enhanced cell proliferation, differentiation, adhesion, and the formation of mineralized tissue. This stark contrast to bioceramics obtained from other fish species, such as tuna or cod, suggests an innovative edge for salmon-based bioceramics [12]. In addition, Venkatesan et al. emphasized that the significant evolutionary gap between humans and fish considerably diminishes the risk of disease transmission, thus further augmenting the appeal of salmon-derived bio-ceramics [13,14,15,16]. Henceforth, in essence, R&D&I has showcased the multi-faceted potential of salmon fish backbone HA, heralding a new era of sustainable molecules and biomaterials that also align with stringent biocompatibility standards, marking a significant stride in the field of regenerative medicine and dentistry, an ongoing *hot* topic in our collaborative laboratories. Indeed, we have recently reported the physico-chemical properties of our *patent-pending* nano-scaled HA material obtained from the backbone of salmon fish [15]. Briefly, our nanoS-HAp is obtained via a modified and innovative alkaline hydrolysis–calcination process that we also optimized for safe and efficient HA extraction. Following the extensive chemical, physical, mechanical, and biological assessments, including X-ray diffraction, electron microscopy, spectroscopy, and the relevant cell viability assays, our optimized extraction and production process(es) yielded salmon-derived HA with a highly crystalline structure, an optimal Ca/P ratio, and excellent biocompatibility. The attractive nano-scale cellular/tissular properties and favorable molecular characteristics—particularly well-suited for simple and complex bone regeneration and repair applications—are comparable to or even surpass those of synthetic, human, bovine, and porcine hydroxyapatite, positioning it as a promising candidate for use in tissue bio-engineering, wound healing, and regenerative medicine indications [15].

The principal aim of the present study is to investigate mechanisms for the efficient and cost-effective laboratory-scaled isolation and extraction of hydroxyapatite from salmon fish bones, yielding workable and clinically malleable biomaterials with osteoinductive potential. This would also contribute to the more sustainable utilization of waste generated by the salmon industry, a prominent Chilean resource. Henceforth, salmon fish bones consist of two primary phases: (1) organic and (2) mineral. The mineral phase is isolated during the HA extraction process. The organic phase of salmon fish bones, primarily composed of proteins, lipids, and other organic compounds, is typically removed during the HA extraction process. This removal is essential to isolate the mineral phase, which is the target for producing high-purity HA for applications in biomaterials. Herein, we conducted distinctive protocols within our participating and collaborative laboratories, drawn from relevant literature [12,14,17,18], for proper comparative purposes. These were then carefully replicated and subjected to lab-scaled characterization via Raman spectrometry followed by X-ray diffraction to both ensure reproducibility and gain initial insights into the physical attributes of the resultant biomaterial, thereby reporting a novel, simplified, efficient, lab-scaled, and *patent-pending* nanoS-Hap prep protocol [15].

## 2. Results

### 2.1. Characterization

**Figure 1:**
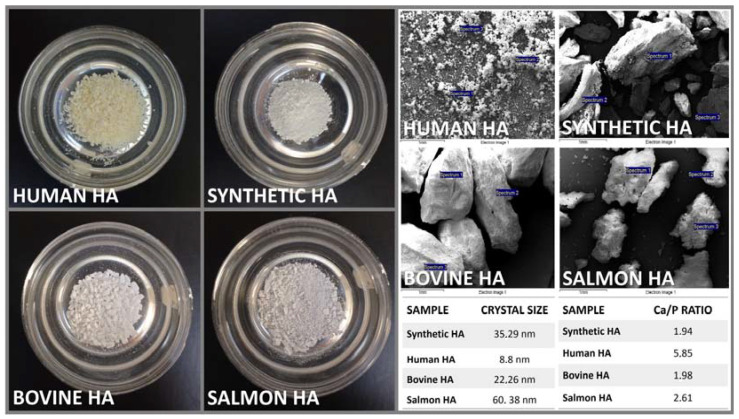
Prepared control and experimental HA samples (**Left**) before characterization and analysis. SEM micrographs of control and experimental HA samples (**Top Right**). Crystal size control and experimental HA samples obtained via XRD and the Ca/P ratio (atomic weight %) for control and experimental HA samples obtained via SEM/EDS (**Bottom Right**). The crystal size, studied using XRD and further analyzed using the Scherrer equation, reported sizes ranging from 8.8 nm to 60.38 nm. Using SEM/EDS analysis, the Ca/P ratio (atomic weight %) for the samples determined the lowest values for our salmon HA and bovine HA with 1.94 and 1.98, respectively, yet these were similar/close to what is often presented in the literature (*the theoretically stoichiometric value of HA was set at 1.67*), validating our experimental protocol. It is noteworthy that all other HA yielded values higher than those commonly reported in the literature. The Ca/P ratio (calcium-to-phosphorus ratio) in terms of the atomic weight percentage (%) is calculated by dividing the atomic weight % of calcium (Ca) by the atomic weight % of phosphorus (P). The atomic weights of Ca and P are approximately 40.08 g/mol and 30.97 g/mol, respectively. As mentioned, human HA has a Ca/P ratio close to 1.67 in its ideal form. Any deviations from this ratio can indicate changes in the bone mineral density and quality.

In the present study, two essential characterization tests, namely Raman spectrometry and X-ray powder diffraction, were employed to assess the obtained salmon fish bone bio-ceramic. The Raman scattering spectroscopy measurements of the HA obtained via mechanical alloying were obtained. Briefly, the Raman spectra were measured using a triple monochromator micro-Raman spectrometer, equipped with a charged coupled device (CCD) detector, and using the 4880 Å exciting line of the Ar-laser. XRD was also performed using an X-ray diffractometer (Phillips X’Pert Pro, Malvern, UK) equipped with a CuKα radiation source set at a 1541 Å wavelength, to analyze the anatomical and molecular structure of the obtained HA crystals. Studies were performed at a current of 30 mA and an accelerating voltage of 40 Kv, over the 2θ diffraction angle range of 2° to 80° using a step size of 0.02°. The patterns obtained were analyzed using Origin pro 2019b software (OriginLab Corp., USA, all mentioned equipment are in our labs). Further, scanning electron microscopy (SEM) and energy dispersive X-ray spectroscopy (EDS) were used to evaluate the Ca/P ratio within samples (Figure 1). Briefly, a Carl Zeiss scanning electron microscope (EVO MA 10, Oberkochen, Germany) equipped with energy dispersive spectroscopy was used to analyze the chemical compositions of the samples. All these analyses were performed at our laboratories within CiiB.

**Figure 2:**
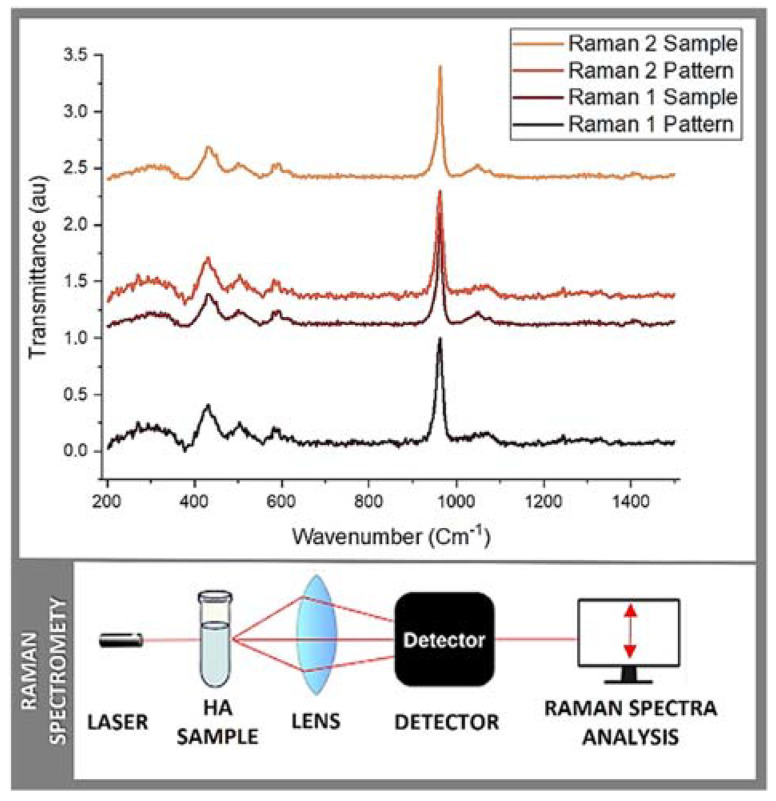
Raman spectrometry: Raman 1 and Raman 2 measurements.

**Figure 3:**
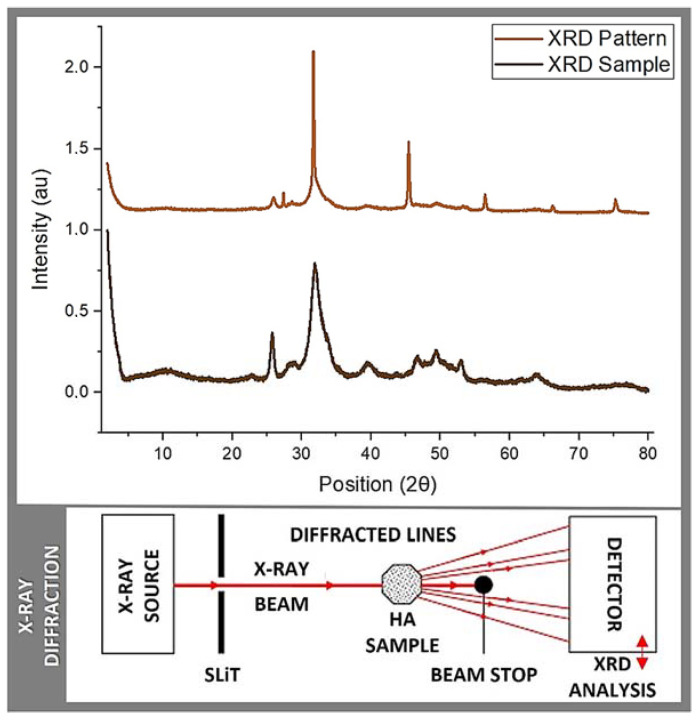
XRD: XRD pattern and sample measurements.

The initial phase of the analysis involved Raman spectrometry (Raman 1), aimed at evaluating the replicability of the material and confirming the presence of hydroxyapatite functional groups (Figure 2). A commercially available synthetic HA sample obtained from Sigma-Aldrich (St. Louis, MI, USA) served as a reference control, for the comparison. This test provided an initial insight into the characteristics of the obtained material while validating the existence of HA functional groups in the control. To ensure the consistency and reliability of the obtained data and findings, the experimental protocol was accurately duplicated, and the resulting sample underwent a secondary round of Raman spectrometry analysis (Raman 2). Additionally, X-ray diffraction (XRD 1) was carried out, using the synthetic HA sample from Sigma-Aldrich as a control for reference (Figure 3). These multiple/repeated characterization tests, to the best of our knowledge, collectively, provided a comprehensive evaluation of the composition and properties of this salmon fish bone HA bioceramic.

### 2.2. Raman Spectrometry

Raman spectrometry is a versatile technique used for the analysis of molecular vibrations in a wide range of materials, from solids to liquids and gases. It is a non-invasive and non-destructive method widely used in research, industry, and various scientific disciplines. It provides valuable information about the chemical composition, molecular structure, and crystal symmetry. Briefly, each chemical bond in a molecule vibrates at specific frequencies, and these vibrations are detected in the Raman spectrum, allowing for chemical identification. Hence, Raman spectroscopy provides insights into the molecular structure and symmetry of a material by revealing details about bond lengths, angles, and the arrangement of atoms within molecules. Further, it can distinguish between different phases of a material, including polymorphs, crystal structures, and amorphous phases. It is useful for materials science and crystallography. Raman spectrometry can also be used to investigate the stress and strain in a material where changes in vibrational frequencies can indicate mechanical stresses within the sample. Last yet not least, it is often employed for quality control assessments and process monitoring in various industries, including pharmaceuticals, food, and bio-materials sciences. Raman spectrometry was principally used in this study to identify the functional groups in the prepared sample(s) in order to establish an approximation of the HA structure and to confirm the calcium phosphate (CaP) basic structure units. The results obtained from Raman 1 and Raman 2 are reported in Table 1 and Figure 2. Briefly, hydroxide (OH), phosphate ion (PO_4_), and carbon trioxide (CO_3_) were present in both batches of samples, as in most CaP. In addition, different bands, such as 623 cm^−1^, 759 cm^−1^, 819 cm^−1^, 845 cm^−1^, and 882 cm^−1^, corresponding to the reference spectra reported in the literature were detected/measured [19,20]. These ranges showed exact coincidences and other significant peaks. The first zone of exact coincidence showed common peak ranges, oscillating between 580 cm^−1^ and 589 cm^−1^, with an average of 580 cm^−1^. This range (i.e., 580 cm^−1^) corresponds to a symmetric P-O stretching vibration (*simultaneous vibration of two bonds, in which the bonds elongate together and contract together*) n4 [19,20], typical of the functional group PO_4_ and characteristic of tetrahedral *biological* apatites [20,21] often reported in the literature [21,22,23]. A second zone of exact coincidence corresponds to a 961 cm^−1^ peak, with the highest spectrum intensity, described as the PO_4_ groups bending mode and PO_4_ vibrations [24]. Associated with this same functional group, a lower intensity peak formed around 1070 cm^−1^–1074 cm^−1^ is described as PO_4_ band stretching vibration [24,25,26]. Additionally, some peaks were present only in a few samples. The obtained peaks of 595 cm^−1^ (Raman1) and 623 cm^−1^ (Raman 2) correlate with 602 cm^−1^–603 cm^−1^ or 632 cm^−1^–635 cm^−1^ belonging to the OH groups [21,23]. Stretching and liberation modes of OH are typical of HA crystallite structures [21,25]. Finally, a 1460 cm^−1^ peak is reported in Raman 2, which could be related to the presence of a CO_3_ group. Research shows that this peak can occur between 1421 cm^−1^ and 1466 cm^−1^. Its presence in bone graft materials is described as a factor that allows the extracellular matrix (ECM), the intricate network of proteins and minerals found in native bone tissue, to be simulated, which in turn would improve osseointegration, biocompatibility, and *early* osseous resorption [26]. Herein, similar to porosity, a critically important factor that allows for the simulation of the ECM whereby controlled porosity is introduced into bone graft materials, it becomes possible to replicate the natural structure of the ECM by serving as a scaffold that promotes cell attachment, proliferation, and differentiation whilst allowing for the ingrowth of blood vessels and the formation of new or de novo bone tissue [27]. Indeed, the presence of this ECM-like porosity in bone graft materials enhances osseointegration and the bone tissue regenerative outcome, as it provides a mechanically supportive and biomimetic environment suitable for the interaction (effective fusion) among the host cells, native bone, and the graft material [26,27,28]. It also improves biocompatibility by mimicking the natural bone micro-environment (more conducive to cell adhesion, proliferation, and differentiation, closely resembling the natural bone micro-environment of the patient) [26,27]. In addition, controlled porosity (intra-channels) can lead to *early* bone resorption, as mentioned above, a desirable feature in bone graft materials, as well as in other regenerative and reparative biomaterials [28,29], as it allows the material to gradually bio-degrade and be substituted by the own bone tissue of the patient during the healing processes [26,27,28,29,30].

### 2.3. X-ray Diffraction

The main objective of X-ray diffraction (XRD) is to determine the atomic and molecular structure (phase) of a crystalline material. XRD helps in identifying hydroxyapatite and distinguishing it from other calcium phosphate phases, such as tricalcium phosphate and dicalcium phosphate, where each phase has a unique diffraction pattern. In this context, XRD is a powerful characterization tool and a widely used analytical technique in materials science, chemistry, and related fields. It involves shining X-rays onto a crystalline sample and observing the resulting diffraction pattern, which is caused by the interference of X-rays scattered by the crystal lattice. Indeed, XRD can determine the crystal composition and structure of a material, which describes the arrangement of atoms or molecules within the crystal lattice. It reveals the unit cell dimensions, atomic positions, and symmetry of the crystal. Moreover, XRD is used to identify the different phases present in a sample, which is crucial for material characterization. It can differentiate between various polymorphs or crystal structures of the same material. Further, XRD can quantify the degree of crystallinity (degree of crystallinity is a measure of the fraction of a material that is crystalline compared to the amorphous or non-crystalline phase. It significantly influences the physical properties of the material, such as mechanical strength, thermal stability, and chemical resistance; i.e., higher crystallinity usually indicates better stability and mechanical strength) in a sample, providing information about the order or disorder of the crystal lattice. XRD also measures the lattice parameters, which include the lengths of the edges of the unit cell (a, b, and c) and the angles between them (α, β, and γ). These parameters are fundamental in defining the crystal structure and can provide significant insights into the properties and behavior of the material/biomaterial. Henceforth, XRD can be used to assess the stress and strain within a crystal lattice, which is important in materials engineering and quality control. Last yet not least, XRD is deemed a vital tool in providing critical information about the size and distribution of crystalline particles in a biomaterial.

In this context, Table 2 and Figure 3 display the results obtained from the conducted quantitative analysis, in which the following spectra were compared: the sample obtained in this study, a JCPDS 74-0565 (*standards compiled by the Joint Committee on Powder Diffraction Standards*) pattern, the sample reported by Shi et al. [12] based on salmon fish bone, and the control sample corresponding to commercially available HA (Sigma-Aldrich). In relation to the data obtained, five bands of transversal coincidence were found for the four spectra, all in the range between 20 and 60 2Theta (2θ; atomic spacing), which belong to the characteristic peaks of CaP (calcium phosphates) that are located at 25, 31, 39, 46, 49, and 53 2Theta.

The obtained spectra have the characteristic defined and sharp shape, proper for crystalline structures. In addition, when contrasting the samples against the Crystallography Open Database (COD) and COD inorganic compounds only (CODI), using Match 3! Software (version 4.0 Build 306), *an easy-to-use software for phase analysis using powder diffraction data and compares the diffraction pattern of your sample to a database*, the presence of Ca, P, and O in different structural combinations was confirmed. The analyzed samples correspond to the essential sub-units of CaP, yet more studies are required to better define their arrangement and the Ca:P ratio. Along with this, traces of elements, such as Sr and/or Mg, were found, potentially beneficial for the biocompatibility of bone graft materials [21,31]. Regarding the conducted qualitative analysis of the samples obtained, the crystal size was measured using the Scherrer equation, which resulted in an average crystal size of 130.1 Å for the bio-ceramics obtained from the salmon fish bone. For the HA control sample (Sigma-Aldrich), an average value of a crystal size of 285.8 Å was obtained. Both crystal sizes, when transformed into nm, corresponded to 13.01 and 25.58 nm, respectively, which are close to the values reported in literature for this type of material [12,13,14,15] (*please re-visit data in* Table 2).

## 3. Material and Methods

The HA extraction and production process involved, primarily, an extensive literature review of articles between the period of 2015 to 2024, wherein the bioceramic extraction process from salmon fish bone (of any type) was described. Henceforth, three published studies [12,14,17] were finally selected to assess the described protocols and if they could be replicated in various facilities at the CiiB of Universidad de los Andes, Santiago de Chile. Modification, customization, and calibration of the final *prep* protocol then followed.

**Figure 4:**
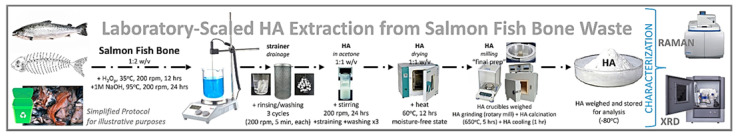
Illustrating the *Prep* protocol for HA extraction from salmon bone waste and analytics. HA is a calcium phosphate compound, Ca_10_(PO_4_)_6_(OH)_2_, that serves as the main inorganic element composition in the bone and teeth. HA extracted from fish bone is considered to be an alternative to synthetic HA from chemicals. Different regions have access to various fish species, leading to the utilization of locally abundant fish waste for HA powder production. These species yield HA with distinct morphologies, porosities, and purities, which makes the choice of the fish source crucial in the process. The selection is often influenced by regional availability, the specific properties of the fish bones, and the goals of sustainability and innovation in material synthesis [31,32]. Fish, such as catfish (*Pangasius hypophthalmus*), cod, tilapia (*Oreochromis* sp.), seabass (*Lates calcarifer*), yellowfin tuna (*Thunnus albacares*), rainbow trout, Whitemouth croaker, and, more recently, red big-eye snapper (*Priancanthus tayenus*), are among the wide range of aquatic or marine animals used in these studies. This growing interest underscores the dual advantage of converting bio-waste into valuable and adaptable HA while addressing environmental pollution challenges. This approach not only promotes sustainable biomaterial synthesis and environmental management, but it also possibly offers cost-effective and eco-friendly processes with a strong potential for industrial-scale applications, providing a greener alternative to traditional HA production methods and protocols [15,18,31,32].

A preliminary bone pre-treatment protocol (to achieve deep cleaning of the raw material; i.e., market/industry-discarded salmon bones) was executed, initiated by submerging the bone in purified water obtained through reverse osmosis (H_2_Op; p for purified) and maintaining it *heated* at a temperature of 35 °C for a duration of 12 h (hrs). Subsequently, the removal process entailed manually extracting the spines and larger voluminous muscle pieces. The vertebrae were then segregated/separated and preserved in storage within the temperature range of −18 °C to −25 °C. This protocol encompassed six sequential steps. Following an adapted and a modified version of Shi et al.‘s protocol (Figure 4), the salmon fish bone underwent separation, thawing, and weighing. A 1:2 weight-to-volume ratio (bone weight to water volume; *w*/*v*) of H_2_Op was heated to 95 °C and stirred at 200 revolutions per minute (rpm) using a magnetic stirrer (Dlab; MS-H-Pro+; Beijing, China).

Once the desired temperature was attained, the bone was then introduced/added, and the specified temperature and stirring conditions were consistently maintained for 1 hr. Subsequently, the liquid was separated through drainage using a fine metal strainer (Ilko^®^; 10 cm Inox. Shunyi District, Chongqing, China). A rinsing/washing step/process was carried out three times in a standard magnetic stirrer with H_2_Op, maintaining the 1:2 *w*/*v* ratio, while continuously/constantly agitating at 150 rpm for 5 min, each time. Following the drainage and removal of the liquid using the strainer, a 1 Molar (M) solution of Merck^®^ sodium hydroxide (NaOH) (Merck is a brand, NaOH provided via a local supplier) was added at the standard 1:2 *w*/*v* ratio and left to react for 24 h, with constant stirring at 200 rpm (alkaline treatment via the use of an alkaline NaOH solution is basically to de-proteinize the bone sample and remove the majority of the organic matter, i.e., proteins). Afterwards, the liquid was once again separated using the described strainer, and the resultant material was subjected to three incessant washing cycles inside the magnetic stirrer with H_2_Op, maintaining a 1:1 *w*/*v* ratio and agitating it at 200 rpm for 5 min in each cycle. The bone was subsequently immersed/submerged in acetone (Sigma-Aldrich, Sigma-Aldrich is globally known, with representatives in countries, so acetone was obtained thru a local vendor) at a 1:1 *w*/*v* ratio and placed on the magnetic stirrer operating at 200 rpm for 24 h. The solution was then decanted with the aid of the strainer and underwent three sequential washing cycles with H_2_Op, carefully preserving the 1:1 *w*/*v* ratio, using a regular yet controlled laboratory bench-top magnetic stirrer rotating at 250 rpm for 5 min, for each cycle.

The resulting material was then subjected to a drying process in an oven (Horizontal flow; WOF-105; Shanghai, China) set at 60 °C for a period of 12 h. The crucibles used in the process were also dried in the oven set at 60 °C for 30 min. Upon reaching/achieving a dry and moisture-free state, the crucibles were weighed and measured using a Shimadzu (AUX 120; Columbia, MD 21046, USA) analytical balance. Subsequently, the grinding procedure was carried out using a rotor mill (Foss; KN 295 Knifetec; Santiago, Chile), consisting of four pulses, each lasting 2 s. Following this, the electric Muffle Furnace (XL-1000c/1200c/1600c; Santiago, Chile) was pre-heated to 650 °C. Once the desired temperature was attained, the sample (i.e., *samples*) was subjected to calcination for 5 h (to ensure that the biomaterial was, free of organic residues), after which it was allowed to cool within the Muffle Furnace for an additional 60 min.

Finally, the resultant final material and sample(s) was/were weighed, labeled, and properly stored in an environment devoid of moisture, maintained at −80 °C and shielded from any exposure to light. It is noteworthy herein that to ensure the reproducibility of the HA extraction procedure, the designed and performed protocol was duplicated on two separate occasions, under the same conditions. The samples were subsequently subjected to Raman spectrometry. This protocol was once again replicated and then underwent a comprehensive characterization through both Raman spectrometry and X-ray diffraction, following the pre-established methodologies [12,13,14,15,16,17,21], manufacturer’s instructions and protocols, and the introduced/applied standardizations at our R&D&I laboratories.

## 4. Discussion

### 4.1. Production

Hydroxyapatite (HA) is a widely used biomaterial in tissue regeneration and repair due to its biocompatibility, bioactivity, and similarity to the mineral component of human bone [1,2,4,6]. Different types of HA can be derived from various sources and through diverse extraction methods, each influencing the characteristics of the extracted and produced HA and its performance, in vitro, in vivo, and in the clinic [1,2,6,8]. Briefly, human-derived HA closely resembles autografts in function, delivering excellent biocompatibility and bioactivity, though its use is limited by availability and ethical concerns. Synthetic HA offers high purity and customization options; however, it may lack the natural bioactivity inherent to biological HAs. On the other hand, whilst bovine- and porcine-derived HAs provide a natural architecture and porosity that support cell growth and tissue integration, they carry potential risks of disease transmission and immunogenicity [1,2,3,4,5,6,8]. Fish-derived HA, such as that obtained from salmon, combines sustainability with desirable nanoscale properties and high bioactivity, positioning it as a strong candidate for bone regeneration [1,2,3,4,5,6,8,9,10,11,12]. Henceforth, each type of HA presents unique benefits and limitations, and the choice of material depends on the specific clinical application, desired properties, and ethical considerations. Table 3 summarizes some of the extraction methods, their characteristics and main observations often reported in the accruing literature.

Today, the realm of osseo-regenerative and -reparative material production has seen substantial R&D&I efforts, with an accruing drive towards developing much more cost-effective and readily available alternatives to better address the persistent challenges of high production costs and storage, amongst others. This pursuit has led to the exploration of novel sources for xenograft-type materials, which have the potential to reduce expenses, enhance biocompatibility, improve cellular and biological interactions, and increase availability. In this context, fish bone-derived xenografts, particularly those sourced from bio-waste, captured the interest of R&D&I groups/laboratories, including our own.

Various methods for producing these xenografts, such as NaOH-based hydrolysis, calcination, and enzymatic hydrolysis, have been reported in the accruing literature. The aim of our present study was to study and describe the process for extracting the mineral phase from salmon fish bones, for the development of a new reproducible protocol based largely on the framework previously proposed by Shi et al. [12], deemed most suitable and practical. Indeed, this protocol focuses on *key* elements, factors and stages designed to eliminate a significant portion of organic matter. This involved the thorough cleaning and removal/elimination of large-scale muscle tissue at elevated temperatures, enzymatic lysis of collagen and proteins in the bone matrix using an appropriate concentration of NaOH, removal of lipids and fats through de-fatting in an acetone solution, and finally, the calcination process to eliminate residual (from previous processes/stages) organic components. Herein, it is crucial to exercise extreme caution to avoid/prevent any damage to the bone mineral matrix, as any remaining residues could potentially alter the structural integrity or act as a vector for contamination [12,13,14,15,16,17,18,21]. As presented in our study, we proposed, designed, and developed an innovative and patent-pending experimental preparation protocol for extracting and producing HA and nano-scaled HA bio-ceramic material from salmon fish bones, which co-integrates alkaline hydrolysis, de-fatting, and calcination processes. This simplified and reproducible approach offers a simple, safe, efficient, reproducible and inexpensive method/protocol for advancing the extraction, formulation and production of novel bone regeneration biomaterials from sustainable and readily available resource(s), possibly impacting the field of functional biomaterials and beyond.

### 4.2. Parametric Analysis

To obtain preliminary insight into the structure of the extracted material, we conducted Raman spectrometry and XRD analyses. Following the initial/primary characterization, we verified the reproducibility of our results, observing a high degree of consistency and satisfactory coherence across the different sample batches. In our Raman spectrometry results, we confirmed the presence of the essential functional groups crucial for the formation of HA or beta-tricalcium phosphate (ß-TCP), such as PO_4_, OH, and CaO_3_. Notably, the characteristic peaks corresponding to these functional groups were consistently detected in all compared samples. To ensure precision and confirm the presence of HA, we performed two sets of comparisons with the selected controls, revealing a very close match. This was particularly evident in the peak with the highest volume, associated with one of the PO_4_ functional group bands. Additionally, the presence of a CO_3_^−2^ group, reported in the 1421 cm^−1^–1466 cm^−1^ peak range (in the Raman 2 set), is linked, in the literature, to enhanced biocompatibility in bone regeneration materials. As a point of reference, herein, we employed synthetic and commercially available HA (Sigma-Aldrich) as a control [19,20,21,22,23,24,25,26,27,30,31,32]. The XRD analysis confirmed the presence of key elements, like Ca, P, and O, as well as trace elements, including Sr and Mg. These trace elements have been identified as potential contributors to the biocompatibility of the regenerative material, consistent with previous research and literature. It is perhaps noteworthy that although we did not find an exact match in the databases (COD and CODI) used, our findings do align with the expected distribution of a crystalline structure and an estimated crystal size, as described in the literature and suitable for osseo-regenerative and -reparative materials of this type [26,27,28,29,30]. Collectively, these results support the further exploration and use of salmon-derived materials for tissue (hard and potentially, soft) repair and regeneration, hereby, indicating their potential advantages over alternatives derived from other fish species and highlighting their practical promise as a valuable resource for the development of new tissue engineering biomaterial(s), an ongoing pre-clinical assay in our labs.

To recap, this study aspired to significantly contribute and advance the sustainability and biocompatibility of biomaterial production through the development of a novel HA derived from Chilean salmon fish bones, a natural resource and biowaste challenge. By leveraging biowaste from the salmon industry, the study not only addresses environmental challenges associated with waste management but also provides a sustainable source of high-quality biomaterial. The extraction/production process is both cost-effective and environmentally friendly, highlighting its potential for large-scale applications. The salmon-derived HA exhibits exceptional functional properties that are critical for the reconstruction of bone tissue defects. The material features a highly crystalline structure, an optimal calcium-to-phosphorus or Ca/P ratio, and excellent biocompatibility. These attributes contribute to its ability to support bone regeneration effectively. The nano-scale cellular and molecular properties of this HA facilitate cell adhesion, proliferation, and differentiation, which are essential for promoting de novo bone formation and integration with existing bone tissue. In today’s clinical indications and applications, this HA can serve as a malleable and effective bone graft material and/or as a supplier (drug delivery vehicle/carrier/system) of peptides, cytokines and growth factors, in/for various therapeutic uses [23,24,25,26,27,28,29,30,32]. Its high bioactivity and favorable molecular characteristics make it an excellent candidate for enhancing tissue bio-engineering, wound healing, and regenerative medicine. By improving the functional performance of bone grafts and regenerative treatments, this material holds potential to significantly enhance patient outcomes and quality of life. Henceforth, integrating this new salmon-derived nano-sized HA into medical practice not only supports sustainable and ethical biomaterial production but also offers a promising solution for addressing simple, as well as complex, tissue repair challenges, ultimately contributing to an improved human health and well-being, aesthetically and functionally.

To summarize, based on the results obtained during this characterization, a microscopic and structural resemblance can be established with other bio-ceramic materials derived from salmon fish bone, as reported in the existing literature. These materials are primarily composed of HA, ß-TCP, or a combination of both. However, to gain a comprehensive understanding of the properties, additional physical characterization tests should be conducted on our produced material. For example, in addition to the conducted SEM recommended to define the surface topography, surface morphology, pore size, and its Ca/P ratio, other microscopy techniques can also be employed. Furthermore, conducting compression tests would provide insights into its compressive strength and compaction potential [12,21,24,26]. In terms of the chemical analysis, thermo-gravimetric analysis can help assess the purity of the sample by quantifying the organic content and moisture. This analysis will be valuable in determining the composition of the final material [12,18,21,24,27,30], a step to be included in our future reports. To evaluate its suitability for biological applications, it is essential to conduct biological tests designed for assessing cyto-/bio-compatibility and efficacy. These tests may include analyses of cell proliferation and alkaline phosphatase activity to determine the ability of the material to support cell proliferation and differentiation, respectively. Such experiments are ongoing in our labs, including pre-clinical in vivo assays using suitable models. Until these thorough assessments are performed, we cannot provide an exact composition or behavior prediction of the material upon clinical/surgical use. However, the lab-scale material we have obtained, under controlled conditions, presents itself as a promising candidate for use in bone tissue repair and regeneration. Its similarities to the various employed control materials, as well as other reported materials in the literature, suggest its potential for such compound application(s).

## 5. Conclusions

Globally, statistics reveal that approximately 2.2 millions of bone graft procedures are conducted annually, with costs estimated at US$ 664 million as of 2021, according to the US-FDA. Furthermore, the number of surgical procedures aimed at repairing bone defects is projected to increase by approximately 13% each year. The *patent-pending* isolated functional biomaterial derived from Chilean salmon fish bones demonstrates promising biochemical properties, including bioactive HA with essential mineral ions. This positions it as a viable candidate for producing laboratory-scaled xenografts suitable for tissue engineering, wound healing, and regenerative medicine, including simple and complex bone repair and regeneration applications, in particular. The described and presented extraction/production process, herein, is straightforward, reproducible, and cost-effective, offering an environmentally sustainable alternative solution to the rising challenge of managing bio-waste from the salmon sector and the broader marine and aquatic industries. Indeed, our modified alkaline hydrolysis–calcination method produced salmon-derived HA with a highly crystalline structure, optimal Ca/P ratio, and excellent biocompatibility. Its nano-scale cellular and molecular properties are particularly well-suited for bone repair and compare favorably to or even surpass those of human, synthetic, bovine, and porcine HA. Further in-depth characterization is key to weigh the full pre-clinical and clinical potential of the biomaterial for/in various bio-medical and -dental applications in tissue regeneration, a focus of the ongoing R&D&I endeavors in our labs.

## 6. Patents

Patent Cooperation Treaty (PCT)—(International) has been filed by the co-authors of this article.

## Figures and Tables

**Table 1:** Sample description and functional group interpretation according to the KnowItAll^®^ HORIBA Edition software (“LabSpec 6 Software Suite”) and Raman database with over 1500 spectra for a comparative analysis, SearchIt^TM^ for database spectral searching, MineIt^TM^ for database visualization and mining ,and Mixture Analysis for individual component(s) ID-ing in a mixture spectrum, through a database search.

Raman 1 Pattern	Raman 1 Sample	Raman 2 Pattern	Raman 2 Sample	HORIBA Scientific Raman Database *
307,572	-	307,572	-	δ(CC) aliphatic chains
-	269,532	-	269,532	δ(CC) aliphatic chains
-	292,969	-	292,969	υ(Se-Se)
-	313,404	-	313,404	υ(Se-Se)
428,881	-	428,881	-	υ(S-S)
-	431,74	-	431,74	υ(S-S)
448,8075	-	448,8075	-	υ(Si-O-Si)
499,951	-	499,951	-	υ(Si-O-Si)
-	502,777	-	502,77	υ(Si-O-Si)
580,40886	581,35	589,707	581,35	υ(C-Cl)
614,708	-	-	623,019	υ(C-I)
727,278	-	-	759,819	υ(C-S) aliphatic
961,748	961,748	961,748	961,748	ν 1 (PO4 3−)/(A/E2)
1049,81	-	1049,81	-	υ(C=S)
1074,87223	1071,03918	1074,87223	1070,33	υ(C=S)
-	1244,80409	-	1244,23	υ(C=S)

* KnowItAll^®^ Informatics System: a spectra database covering many applications, available for data mining and analytical and comparative studies, from HORIBA Scientific, Kyoto, Japan.

**Table 2:** XRD spectra of bio-ceramics obtained from salmon fish bone compared with the spectra of synthetic HA (Sigma-Aldrich) and the spectra reported in JCPDS 74-0565 and Shi et al. [12], respectively.

JCPDS 74-0565 *	Shi et al. [12] Natural HA	HA Sigma Aldrich	Salmon Fish Bone Bio-Ceramic
-	-	10.8	10.43
25.882	25.845	25.81	25.9
-	-	28.08	28.37
-	-	28.89	28.5
-	-	29.64	29.16
31.765	31.792	31.73	31.6
32.194	32.142	32.13	-
32.896	32.935	32.86	-
34.062	34.055	34	-
39.79	39.816	39.74	39.46
-	-	45.25	45.41
46.693	46.698	46.61	46.72
-	-	48.01	48.04
49.489	49.496	49.39	49.42
50.474	50.568	50.42	-
-	-	51.21	51.41
53.218	53.183	53.1	53.27
-	-	57.8	56.43
-	-	62.93	63.84
-	-	66.26	66.17
-	-	75.49	75.25

* JCPDS 74-0565: Joint Committee on Powder Diffraction Standards.

**Table 3:** HA is a widely used biomaterial in tissue regeneration due to its biocompatibility, bioactivity, and similarity to the mineral component of human bone. Different types of HA can be derived from various sources and via diverse extraction methods, each influencing the characteristics of the extracted and produced HA and its reported performance in the available and accruing literature.

HA	Extraction and/or Production Method	Main Characteristics	Main Effects in Pre-Clinical Studies
Human-derived	Auto-/Allo-graft obtained from human donor bone, typically through demineralization, sterilization, and sometimes freeze-drying to produce a bone graft material.	- Highly similar to the patient’s own bone in composition and structure. - Contains natural bone matrix proteins that may enhance osteo-induction.	**in vitro**: Supports robust cell attachment and differentiation, often better than synthetic or animal-derived HA due to its bioactive matrix. **in vivo**: Excellent biocompatibility and osteointegration, with reduced risk of immune rejection. Yet, availability and ethical considerations limit its use.
Synthetic	Synthesized through chemical precipitation, sol-gel processes, hydrothermal methods, and other wet chemical techniques.	- High purity and controlled particle size. - Tailorable crystallinity and porosity depending on the synthesis conditions. - Generally, lacks the organic components found in natural HA.	**in vitro**: Excellent biocompatibility, supports cell attachment and proliferation. Bioactivity can vary depending on the crystallinity and surface area. **in vivo**: Often shows good integration with host tissue, but may have slower resorption rates compared to natural HA. Absence of organic components may reduce its osteoinductive potential.
Bovine-derived	Derived from bovine bone through calcination or enzymatic treatment to remove organic components while preserving the mineral phase.	- Naturally occurring HA structure with some residual organic matrix. - High porosity and similar architecture to human bone.	**in vitro**: Promotes cell attachment and differentiation. Natural porosity enhances nutrient exchange. **in vivo**: Shows good osteoconductivity and integration, but there may be concerns regarding disease transmission and immune response, although these are typically minimal after proper processing.
Porcine-derived	Similar to bovine HA, obtained through thermal or chemical processing of porcine bone to isolate the mineral phase.	- Comparable to bovine HA in terms of structure and composition. - May have slightly different mineral content and porosity due to species-specific differences.	**in vitro**: Supports cellular activities, such as adhesion, proliferation, and differentiation. **in vivo**: Demonstrates good biocompatibility and osteoconductivity, but similar to bovine HA, it may present a risk of immunogenicity or disease transmission.
Fish-Derived	Extracted from fish bones (e.g., Chilean salmon) through processes, such as alkaline hydrolysis, calcination, or enzymatic treatment.	- Contains a highly crystalline mineral phase with a favorable Ca/P ratio. - Often exhibits nano-scale features and a higher surface area compared to mammalian sources.	**in vitro**: Excellent biocompatibility, promoting cell adhesion and proliferation. The nano-scale structure may enhance bioactivity and osteoinductive potential. **in vivo**: Demonstrates promising osteoconductivity and integration with host tissue. The sustainable sourcing from fish waste offers an eco-friendly alternative to traditional sources.

## Data Availability

All raw data underlying the results are included herein, and no additional source data are required.
